# Strategic use of Big Data: implementing reference intervals for serum folate and serum cobalamin

**DOI:** 10.11613/BM.2025.010705

**Published:** 2025-02-15

**Authors:** Alicia Madurga, Ariadna Arbiol-Roca, Maria Rosa Navarro-Badal, Anna Cortes-Bosch de Basea, Dolors Dot-Bach

**Affiliations:** Territorial Clinical Laboratory Metropolitan South, University Hospital Bellvitge, Barcelona, Spain

**Keywords:** biochemistry, cobalamin, folate, indirect method, reference intervals

## Abstract

**Introduction:**

Defining trustworthy reference intervals (RIs) for serum folate (FOL) or serum cobalamin (VITB12) is a difficult task. The purpose of this study is to use an indirect approach from the laboratory information’s system to indirectly generate RIs for FOL and VITB12.

**Materials and methods:**

A retrospective observational study was performed at a tertiary-care laboratory’s hospital during 12 months. All FOL and VITB12 tests were measured using a Cobas8000 e801 system (Roche Diagnostics, Mannheim, Germany). The RIs were calculated using a non-parametric approach. The RIs established in the present study were verified by calculating the fraction of RIs that fell outside the new RIs, in two validation cohorts sampled using the direct and indirect method.

**Results:**

A total of 19,214 (FOL) and 27,420 (VITB12) results were obtained. The RIs were 4.5 nmol/L (90% confidence intervals (CI) 4.4-4.6) to 38.4 nmol/L (CI 38.3-38.5) for FOL and 140 pmol/L (CI 139-141) to 659 pmol/L (CI 657-660) for VITB12. The verification included 8,798 FOL results and 7,365 VITB12 results. For both magnitudes was acceptable since only 0.1% of FOL and 0.02% of VITB12 results fell outside the RIs. Finally, the RIs were verified using a direct method with twenty individuals. For FOL 20/20 cases and 19/20 of VITB12 cases fell within the estimated RIs.

**Conclusions:**

In summary, the use of an indirect data approach has enabled us to calculate RIs for FOL and VITB12. The RIs obtained in our study are lower than those proposed by the manufacturer for both FOL and VITB12.

## Graphical abstract

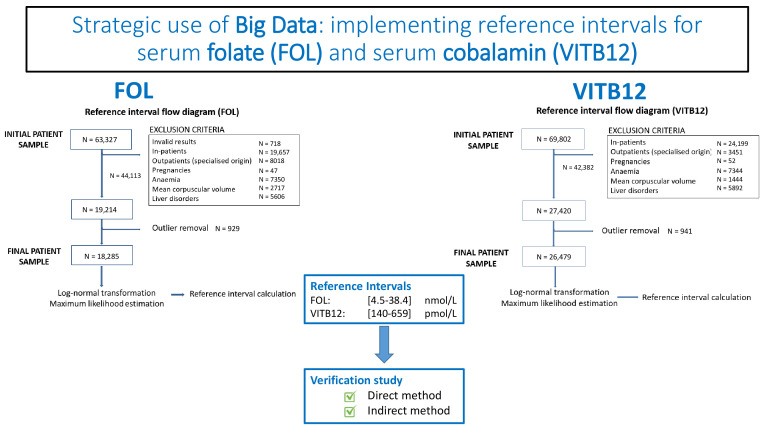

## Highlights

• The reference intervals of serum folate provided by the manufacturer classify many patients with low values in an apparently healthy population tested in our laboratory

• New reference intervals created by an indirect method for serum folate and cobalamin could apply to laboratories that have a similar general population and use the same Roche assay

• The indirect approach reduces the workload and cost of establishing reference intervals

• It is a cheap, specific and rapid method

## Introduction

Cobalamin (VITB12) and folate (FOL), also known as vitamin B12 and vitamin B9, belong to the group of compounds called B-complex vitamins, which are classified as hydro-soluble vitamins (*1*). Factors such as malnutrition, intestinal malabsorption, and veganism may result in the inadequate absorption of some of these micronutrients (*1*).

VITB12 and FOL deficiency may lead to different clinical disorders, including megaloblastic anemia, irreversible neuropathy, neuropsychiatric symptoms, and DNA damage due to a higher level of incorporation of uracil into genomic DNA (*2*-*4*). Poor maternal folate status is also associated with congenital anomalies of the brain, *abruptio placentae*, preeclampsia, and preterm delivery (*5*). A disruption of the tetrahydrofolate and 5-methyltetrahydrofolate generation cycles leads to hyperhomocysteinaemia, which has been linked to various diseases, including vascular and coronary diseases (*3*).

Some guidelines recommend testing for VITB12 only in patients who are at risk of deficiency; there is no evidence to support routine screening for asymptomatic patients (*6*). It is also not recommended to use FOL testing for screening – it has been agreed upon that focusing only on patients who are at risk of deficiency ensures the cost-effectiveness of testing (*7*). If a patient has risk factors but is asymptomatic, then it may be worth considering supplementation with both VITB12 and FOL without testing the patient (*6*).

Generally, the determination of these biomarkers uses automated immunoassays performed using serum samples. Elecsys Folate III and Elecsys Vitamin B12 II are competitive immunoassays provided by Elecsys immunoassay systems (Roche Diagnostics, Mannheim, Germany).

Several years ago (2016), some manufacturers introduced re-standardised versions of the assays in accordance with the World Health Organization (WHO) International Standard-National Institute for Biological Standards and Control (NIBSC) code 03/178 (*8*).

Additionally, in 2016 the supplier of our measuring system (Roche Diagnostics, Mannheim, Germany) introduced a re-standardised version of their third-generation folate assay (Elecsys Folate III), which is traceable to standard folate WHO IS 03/178, therefore requiring a new estimation of the reference intervals (RIs). Since the introduction of this new assay there has been a significant increase in the number of patients with FOL results below the lower limit of the reference range in our laboratory, which is a finding that has been supported by others (*8*).

There are differences between FOL RIs based on published results, and single cutoff values are not well established (*4*, *9*).

Moreover, fortified food consumption due to regulatory policies or supplement intake should be taken into account (*10*).

Appropriate RIs are essential tools for diagnosing deficiencies of these vitamins, as the broad range of non-specific symptoms in subclinical deficiencies complicates their clinical diagnosis (*3*, *11*).

The classic approach to defining RIs is the “direct” method, which is recommended by the Clinical and Laboratory Standards Institute (CLSI) (*12*). It employs a direct-sampling method for a population recruited using inclusion and exclusion criteria.

Nevertheless, “indirect” approaches such as those using big data, which rely on hospital’s existing data, are becoming an increasingly attractive option for many clinical laboratories (*13*-*15*). The International Federation of Clinical Chemistry and Laboratory Medicine (IFCC) advises using the indirect approach as a different option for establishing reference intervals in local laboratories and verifying existing intervals from direct studies or kit inserts (*12*, *16*).

This study describes the determination of RIs for both FOL and VITB12 using an indirect approach. Furthermore, the RIs obtained were validated with a second verification performed by the indirect approach, and following the guidelines of the CLSI EP28-A3c using several reference individuals by the direct method (*12*).

## Materials and methods

### Study design

**Figure 1:**
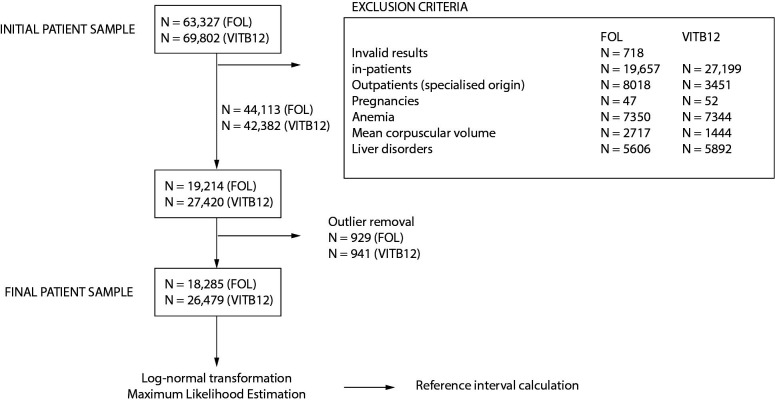
Reference interval flow diagram for serum folate (FOL) and serum cobalamin (VITB12). A flow diagram summarising the steps that are taken to obtain the final data in order to calculate the reference intervals for FOL and VITB12.

A retrospective observational study was performed at a tertiary-care university hospital during 12 months (January-December 2022). Laboratory results for FOL and VITB12 were collected from our Laboratory Information System (LIS) (Infinity/OMNIUM, Roche Diagnostics, Mannheim, Germany). A total of 63,327 FOL results and 69,802 VITB12 results were extracted from the LIS (Fig.1).

Only patients coming from primary care (both adults and pediatric populations) were included in the database.

This work has been carried out following the Code of Ethics of the World Medical Association (Declaration of Helsinki) for experiments involving humans. The manuscript has been approved by the hospital’s Clinical Research Ethics Committee (PR348/23). An appropiate informed consent was obtained from each research participant.

The description of the exclusion criteria applied to the database before processing the initial data is summarized in Table 1.

**Table 1:** Description of the exclusion criteria applied to the database before processing the initial data

Individuals with laboratory results:	Male	Female
Mean corpuscular volume	> 98 fL	> 98 fL
Hemoglobin	< 130 g/L	< 120 g/L
Albumin	< 35 g/L	< 35 g/L
gamma-glutamyltransferase	> 67 U/L	> 30 U/L
In-patients		
Pregnant women		
Requests from all medical departments except primary care and pediatrics		
Patients in diagnosis or follow-up protocol for liver disease and anemia		
Multiple measurements of FOL or VITB12 in one individual		
Patients with duplicate analyses		
Patients with hemolyzed samples		
FOL - serum folate. VITB12 - serum cobalamin.		

To verify the RIs, we used both the indirect and direct method. For that purpose, a new set of data was collected from the LIS (January-March 2023) and it was used to verify the newly estimated RIs for FOL and VITB12. A second verification of the RIs was performed using a direct method. Following the CLSI EP28-A3 guideline, FOL and VITB12 were measured in a group of 20 apparently healthy individuals (*12*). All volunteers met the inclusion criteria (Table 1). They had no history of chronic disease, and none of them were taking any kind of vitamin supplements.

### Methods

Venous blood samples were collected in serum tubes (BD Vacutainer SST II Advance ref. 366468, Becton, Dickinson and Company, Franklin Lakes, USA). The samples were centrifuged at 1500xg for 10 minutes. FOL and VITB12 serum concentrations were measured using an electrochemiluminescence immunoassay (ECLIA) an automated Cobas 8000 e801 system analyzer (Roche Diagnostics, Mannheim, Germany). The Roche Diagnostics Elecsys Folate III Assay (ref. 07027290190), and the Roche Elecsys Vitamin B12 II Assay (ref. 07028121190) were employed.

The measurement range for the Elecsys Folate III Assay is 2.72-45.4 nmol/L, and the limit of quantification (LOQ) is 4.54 nmol/L. Regarding the Elecsys Vitamin B12 II Assay, the measurement range is 73.8-1476 pmol/L, and the LOQ is 111 pmol/L.

Internal quality control was correct for both FOL and VITB12 using the Liquicheck Immunoassay Plus Control (ref. 361, 362 and 363, Bio-Rad, Hercules, United States). The coefficient of variation for FOL was 10.3%, 12.6% and 10.1% and for VITB12 was 8.6%, 5.2% and 4.8% for levels 1, 2 and 3 respectively (the maximum allowable coefficient of variation was 17.8% for FOL and 13.3% for VITB12). All external quality controls were acceptable during the year of data extraction.

### Statistical analysis

The statistical model suggested by Harris and Boyd and recommended by the IFCC was used for the assessment of possible differences between age and sex, in which the means and standard deviations of the subgroups are considered as a separate different standard deviation that may produce different limits (*12*, *17*). Differences of partition were studied based on both sex and age (< or > 18 years old). The Kolmogorov–Smirnov test was used to examine whether the variables were normally distributed. The exclusion criteria were applied, and any outliers were removed according to the Tukey method using the first quartile (Q1), the third quartile (Q3), and the interquartile range (IQR) (*18*). The lower and upper cutoff values for outliers were identified as Q1 - 1.5IQR and Q3 + 1.5IQR, respectively. The Maximum Likelihood Estimation method (MLE) was employed (*19*).

The lower and upper reference limits for each variable were estimated using the 2.5th and 97.5th percentiles of the distribution test results with a 90% confidence interval (CI).

With respects to the verification using an indirect method, the same statistical approach was performed as in the first study. The RIs were verified if 5% or less of the patient’s values fell outside the RIs previously established. Regarding the verification using a direct method, the reference limits that have been established may be considered valid if no more than two of the twenty tested subjects’ values (or 10% of the test results) fall outside of those original reported limits.

Statistical analysis was performed using the following software: R statistics, R studio (R Core Team, New Zealand), and Excel- Analyse it 2010 (Microsoft, Redmond, USA).

## Results

After applying the exclusion criteria (Table 1), the data included 19,214 FOL results and 27,420 VITB12 results. The distribution of the data was non-parametric. The elimination of outliers by applying ± 1.5 IQR meant that the final data included 18,285 reference individuals for FOL and 26,479 reference individuals for VITB12 after the elimination of 929 and 941 values, respectively, from the data sets. Table 2 shows the description of subjects that were included in this study. In total, there were more women, who accounted for 12,226 of the FOL measurements and 16,921 of the VITB12 measurements. The median patient age was 59 (1-103) years old for FOL and 63 (1-106) years old for VITB12 assessments.

**Table 2:** Reference individuals’ description and reference intervals summary for serum folate and serum cobalamin

**Parameter**	**FOL**	**VITB12**
N	18,285	26,479
male	6059	9558
female	12,226	16,921
Median age (years) (min-max)	59 (1-103)	63 (1-106)
Median	13.1 nmol/L	310 pmol/L
Lower Reference interval (90% CI)	4.5 (4.4-4.6) nmol/L	140 (139-141) pmol/L
Upper Reference interval (90% CI)	38.4 (38.3-38.5) nmol/L	659 (657-660) pmol/L
FOL - serum folate. VITB12 - serum cobalamin. N - number of reference individuals. CI - confidence interval.		

Following Harris and Boyd criteria, differences of partition were studied based on both sex and age (< or > 18 years old) (*17*). The results showed that no partition should be made (s1/s2 > 1.5 or z ≥ z*) for both sex (s1/s2 = 1.08; z = - 13.6; z* = 44.3) and age (s1/s2 = 1.1; z = - 13.9; z* = 44.7).

**Figure 2:**
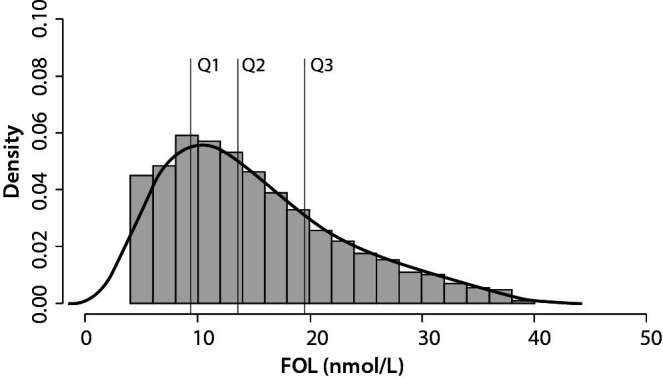
Distribution histogram and density plots of the serum folate concentrations. FOL - serum folate. Q - quartile.

**Figure 3:**
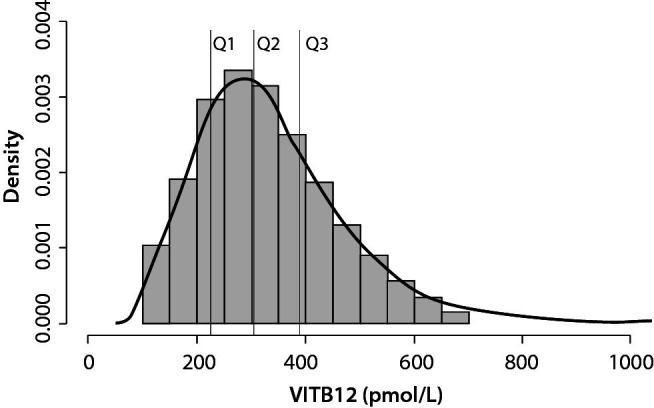
Distribution histogram and density plots of the serum cobalamin values. Q - quartile. VITB12 - serum cobalamin.

The RIs calculated using a non-parametric method for both FOL and VITB12 are summarised in Table 2. The RIs were 4.5 nmol/L (CI 4.4-4.6) to 38.4 nmol/L (CI 38.3-38.5) for FOL and 140 pmol/L (CI 139-141) to 659 pmol/L (CI 657-660) for VITB12. The distribution histograms for FOL and VITB12 are shown in Figures 2 and 3, respectively.

The newly established RIs were validated using an indirect verification method. The verification included 8798 FOL results and 7365 VITB12 results. Regarding the subjects, there were 5681 women and 2603 men for the FOL measurements, and 4851 women and 2505 men for the VITB12 mesuraments. The median patient age was 58 (1-101) years old for FOL and 58 (15-101) years old for VITB12 assessments. The verification was considered valid if ≤ 5% of the results fell outside the RIs. For both magnitudes was acceptable since only 0.1% of FOL and 0.02% of VITB12 results fell outside the RIs.

Finally, the RIs were verified using a direct method with twenty individuals. The mean age was 47 (21-67) years, accounting for 14 women and 6 men.The mean serum concentrations of FOL and VITB12 were 15.8 (4.8-34.6) nmol/L and 336 (176-771) pmol/L, respectively. FOL and VITB12 assay findings in both instances fell within the two limits established in our study (lower and upper RIs); specifically, 20/20 of FOL cases and 19/20 of VITB12 cases fell within these limits.

## Discussion

The FOL RIs estimated in this study (4.5-38.4 nmol/L) is comparable to the RIs estimated in another study that was performed in a similar population using a direct method, thus supporting our chosen approach (*11*). Other European groups have previously published FOL RIs using the recalibrated Elecsys Folate III Assay. In a study performed in the UK, Hepburn *et al.* found similar results to those from our group; in their review, they used a large dataset of FOL values measured in an adult population as prescribed by general practitioners, and the estimated FOL RI was 5.4-39.7 nmol/L (*8*). A German report from Evliyaoglu *et al.* estimated the FOL RI as 4.3-43.3 nmol/L, which was also similar to our results (*20*). On the other hand, Vos *et al.*, in a report performed on a general Dutch population, proposed an FOL RI of 7.3-38.5 nmol/L, irrespective of age, which is higher than our results and those of the other studies (*10*).

Separate RIs for men and women or for different age groups may not be justified unless they are clinically useful or physiologically relevant. According to the Harris and Boyd criteria, no significant differences between gender or age were found (*17*). Thus, neither FOL nor VITB12 required specific RIs according to gender or age.

It also should be noted that in all studies, including ours, the estimated FOL RIs were lower than those suggested by the manufacturer (8.8 to 60.8 nmol/L) that were established with a population of an age of 20-65 years old. According to the assay description, the Elecsys Folate III Assay’s measurement range is 4.54-45.4 nmol/L. This measurement interval is remarkably inadequate, especially at the lower range of measurement, and it has been proven in our study and others that the limit of quantification (LOQ) is insufficient to cover the interval of all healthy individuals (*9*). This technical limitation hinders the ability to properly establish cutoff values in cases of FOL deficiency.

Thus, it should be questioned whether cases of FOL deficiency could be evaluated using the 2.5th percentile, which depends on the specific measurement interval, considering that the threshold of FOL deficiency should not vary between people. To overcome the difficulty of working with values lower than the threshold of < 4.5 nmol/L (that is, at the 2.5th percentile), the MLE tool allowed us to make robust estimates with censorship data.

Regarding the RI for VITB12, our measures, ranging 140-659 pmol/L, showed a wider interval than the one proposed by the manufacturer (145-569 pmol/L). Nonetheless, if we compare our RI to those of other studies, then we find that Solé-Enrech *et al.* reported a lower RI (111-513 pmol/L) when studying a similar population using a direct method (*11*).

Following previous reviews, we verified our RI results for FOL and VITB12 with another cohort of data that was extracted over the course of three months (*13*, *21*). No differences were found between the two studies, accounting for 0.02% of patients with values that fell outside of the FOL RI range and 0.1% of patients with values that fell outside of the VITB12 RI range. Later, we also confirmed our RIs using the traditional direct method by studying twenty healthy individuals. All of their measures fell within the newly calculated RI ranges.

Thus, this data underscores the advantages of establishing RIs using an indirect approach compared to a direct method. This data is also in line with recent reports that used the direct method. Moreover, we used two alternative methods to validate our results. One of them was performed following the recommendations of the CLSI document EP28-A3c, and the other was based on the indirect method, where we used a second data set from an external database (*12*).

When interpreting this data, it is clearly important to remember that some clinical data was not available. The criteria used to select individuals were applied using laboratory tests ordered by general practitioners.

A limitation of this study was that we could not apply exclusion criteria if the individuals were consuming vitamins or fortified foods. Therefore, given that the disease status for both vitamins is related to their deficiency, we would advise defining a cutoff using only the lowest reference values for laboratory reports: FOL ≥ 4.5 nmol/L and VITB12 ≥ 140 pmol/L.

The data presented here did not address the homocysteine concentrations of patients because primary care physicians cannot request this from the portfolio of laboratory services. This is most likely a relevant line of investigation, given that the accumulation of homocysteine is linked to a disruption of the tetrahydrofolate and 5-methyltetrahydrofolate generation cycles.

According to the British Journal Guidelines for the diagnosis and treatment of VITB12 and FOL disorders, the clinical picture is of the most importance when assessing the significance of test results (*22*).

Individuals at risk for VITB12 deficiency (*e.g.* strict vegan, bariatric or gastric surgery, and lactation or pregnancy with limited intake of foods from animals) should receive oral VITB12 supplements. Individuals at risk for FOL deficiency (*e.g.* chronic hemolytic anemia, chronic alcohol use, malnutrition) should receive folic acid supplementation.

There is no “gold standard” test to define VITB12 deficiency. Both VITB12 and FOL are first-line tests of choice to assess both deficiencies. If there are discordances between test results and strong clinical features of deficiency, prompt treatment should be recommended. Definitive cutoff points to define clinical and subclinical deficiency states are not possible, given the different methodologies used, and technical issues, therefore local RIs should be established.

Thus, in summary, the use of an indirect data approach has enabled us to calculate RIs for FOL and VITB12. The RIs obtained in our study are lower than those proposed by the manufacturer for both FOL and VITB12.

This data highlights the fact that the new RIs created by an indirect method could apply to laboratories that have a similar general population and use the same Roche assay. It is a very promising approach as it lessens the workload and costs of the RIs establishment process and it is a cheap, specific, and fast methodology.

## Data Availability

Data are available from the authors upon reasonable request and with permission of (third party).
